# MetaSEM: Gene Regulatory Network Inference from Single-Cell RNA Data by Meta-Learning

**DOI:** 10.3390/ijms24032595

**Published:** 2023-01-30

**Authors:** Yongqing Zhang, Maocheng Wang, Zixuan Wang, Yuhang Liu, Shuwen Xiong, Quan Zou

**Affiliations:** 1 School of Computer Science, Chengdu University of Information Technology, Chengdu 610225, China; 2 Institute of Fundamental and Frontier Sciences, University of Electronic Science and Technology of China, Chengdu 610051, China

**Keywords:** meta-learning, gene regulator network inference, structural equation model, bi-level optimization

## Abstract

Regulators in gene regulatory networks (GRNs) are crucial for identifying cell states. However, GRN inference based on scRNA-seq data has several problems, including high dimensionality and sparsity, and requires more label data. Therefore, we propose a meta-learning GRN inference framework to identify regulatory factors. Specifically, meta-learning solves the parameter optimization problem caused by high-dimensional sparse data features. In addition, a few-shot solution was used to solve the problem of lack of label data. A structural equation model (SEM) was embedded in the model to identify important regulators. We integrated the parameter optimization strategy into the bi-level optimization to extract the feature consistent with GRN reasoning. This unique design makes our model robust to small-scale data. By studying the GRN inference task, we confirmed that the selected regulators were closely related to gene expression specificity. We further analyzed the GRN inferred to find the important regulators in cell type identification. Extensive experimental results showed that our model effectively captured the regulator in single-cell GRN inference. Finally, the visualization results verified the importance of the selected regulators for cell type recognition.

## 1. Introduction

The inference of gene regulatory networks (GRNs) allows for a better understanding of transcriptional regulation and how it works in cell-type identification. The models of GRN inference based on scRNA-seq achieved success in cancer treatment [1], the recognition of cellular homeostasis [2], and single-cell multi-omic studies [3]. However, single-cell RNA sequencing technology has many limitations, such as technical noise [4], high gene variability [5], and batch effect [6]. Those regulators whose activities are highly variable among different cell types and predict a small set of essential regulators for significant cell types still need more attention. Therefore, inferring gene regulatory networks to study cell-specific phenomena on computational methods is a challenging problem in bioinformatics.

Recently, deep learning has brought new solutions [7,8] to single-cell GRN inference based on coexpression [9]. The supervised approach has two ways to infer GRNs. One directly sets a determined ground-truth label as the model convergence target, such as DGRN [10] and DeepDRIM [11]. The other is to embed a ground-truth network into the model, such as the GRGNN [12] and scSGL [13]. These models could predict the potential gene regulatory relationships in high-dimensional scRNA-seq data. However, the supervised method can only be applied to general tasks with label data. The unsupervised approach facilitates GRN inference without a ground-truth label, such as VEGA [14] and DeepSEM [15]. SCODE [16] is a machine learning algorithm based on linear ordinal differential equations. GENIE3 [17] and GRNBoost2 [18] can also complete GRN reasoning without the label. However, the problem of highly sparse data features still needs to be resolved better.

Meta-learning is adept at solving parameter initialization and insufficient data labels when using small-scale samples [19]. The meta-learning model is composed of a basic learner and a meta-learner. The basic learner extracts feature information through representation study. The meta-learner guides the basic learner to complete training tasks by learning the parameters of the basic learner. The meta-learner synthesizes the training experience of all modules and provides the initial parameters for new task training. By introducing meta-learning, the generalization ability of deep learning models can be improved to solve the highly sparse data features. For example, Kun Fu et al. employed transfer learning to alleviate the training problem of meta-learning on few-shot tasks [20]. Arkabandhu Chowdhury et al. proposed a meta-learning method to complete the data classification on small-scale samples [21]. Zitian Chen et al. solved the low data samples of one-shot learning by meta-learning in the image deformation problem [22]. However, the existing meta-learning methods ought to sufficiently adapt to the single-cell GRN inference task.

**Figure 1:**
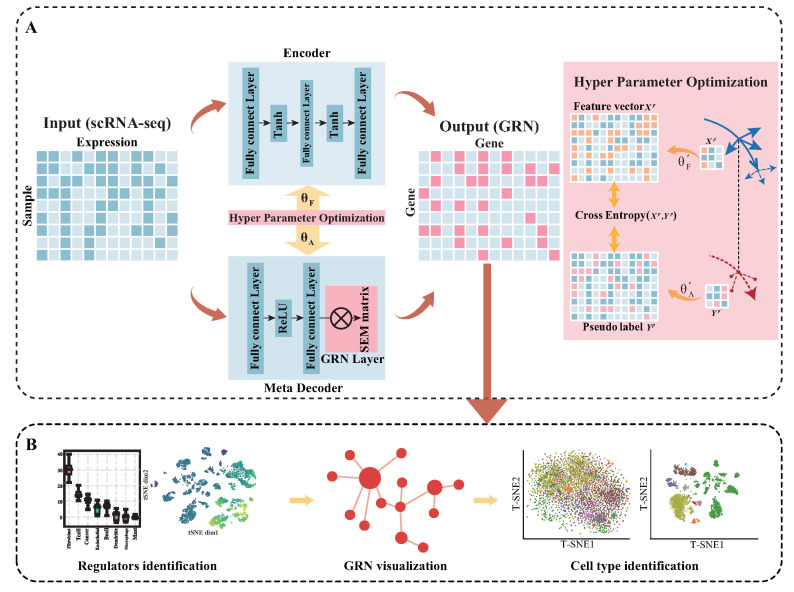
(**A**) The overview of MetaSEM: the meta-decoder extracts the regulatory relationship to output a pseudo-data label. The encoder transforms the data feature into feature vectors. The GRN Layer is a specially designed layer for embedding the SEM matrix. The red arrows indicate the outer loop, and the yellow arrows indicate the inner loop. With hyperparameter optimization, MetaSEM integrates the outer and inner loop based on gradient. The $\theta_{F}$ represents the hyperparameters of the encoder, and $\theta_{A}$ represents the hyperparameters of the meta-decoder. (**B**) By analyzing the SEM matrix, MetaSEM performs three major functions: identification of the regulators, GRN visualization, and cell-type identification.

To address the problems above, we proposed a meta-learning framework called MetaSEM (Figure 1) to infer GRN from scRNA-seq. Specifically, we adopted meta-learning to optimize the parameters of each module for learning high-dimensional data features. Next, we employed a meta-decoder to provide pseudo-data labels for the encoder. Then, we put the feature vectors extracted from the encoder into the training process. After that, considering the gene regulatory relationship is an endogenous variable and the gene expression information is an exogenous variable, we initialized a structural equation model (SEM) [23] adjacency matrix as the GRN layer. We considered the matrix as the regulatory weight matrix and embedded it into the meta-decoder. Finally, all the parameters were optimized using a Bi-Level optimization.

In this work, we verified the reliability of MetaSEM from the following aspects. We first compared the model performance of MetaSEM with several state-of-the-art methods. The experimental results showed that MetaSEM was significantly better than current methods in terms of EPR, AUPR, and AUROC. Next, to analyze the robustness of MetaSEMwe, we explored essential data features in single-cell data at different data scales. We then generated cell-type-specific GRNs on the bulk RNA-seq dataset for further research. Pearson correlation analysis of GRNs and gene expression data analysis indicated that the GRNs were cell-type-specific. Finally, the visualization of cell-type-specific GRNs within the HNSCC dataset demonstrated the regulators’ importance in identifying cell types.

## 2. Results and Discussion

### 2.1. Comparison with Existing Methods

To verify the performance of MetaSEM, we compared the model with four methods: DeepSEM [15], DGRN [10], GENIE3 [17], and PIDC [2] on the BEELINE dataset. As shown on the 1000 gene datasets in Table 1, MetaSEM outperformed the existing methods on the three evaluation metrics. The EPR of MetaSEM in mHSC-L, mHSC-G, and mHSC-E datasets were 1.36, 1.41, and 1.21, respectively. The EPR of MetaSEM in the mHSCs dataset was 0.15 higher than that of DeepSEM on average. MetaSEM was 0.41 higher than DGRN in AUPR and AUROC. In addition, GENIE3 and PIDC are far inferior to deep learning methods because they are unsupervised machine learning methods. The results show that MetaSEM can effectively memorize gene regulatory relationships and take such relationships to guide the model to extract essential information.

To further evaluate the performance of MetaSEM, we trained MetaSEM on the 500 gene datasets. It should be noted that the label data in 500 gene datasets was just as 30% of that in 1000 gene datasets. The results on the 500 gene datasets in Table 1 show that MetaSEM still surpassed the other methods. The average performance of the model on the three metrics was 1.34, 0.78, and 0.75. As a result, we can observe that MetaSEM can perform well in the shortage of label data.

### 2.2. MetaSEM Can Adapt to High Dimensions and Sparse Characteristics

**Figure 2:**
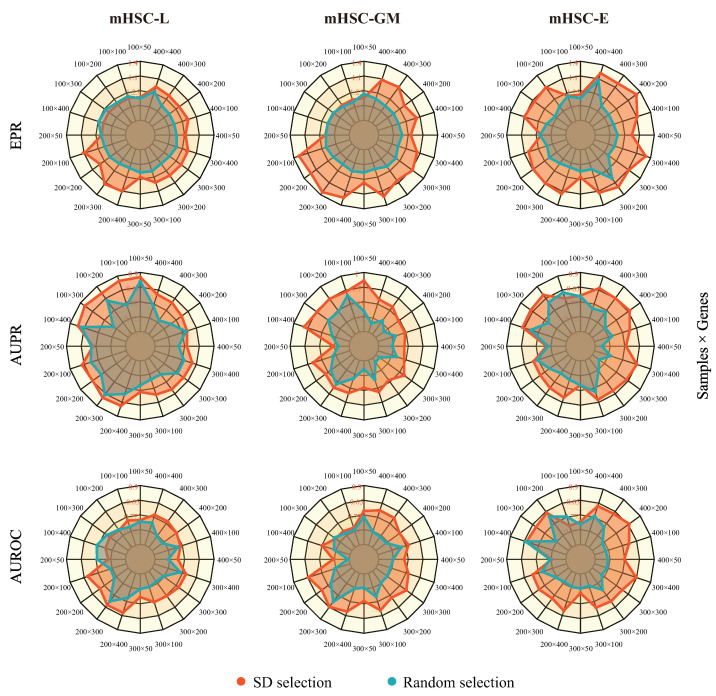
The robustness of our model on different data scales. Each column corresponds to a cell’s sub-dataset (**left**: mHSC-L, **middle**: mHSC-GM, and **right**: mHSC-E), and each row corresponds to an evaluation index (**top**: EPR, **middle**: AUPR, and **bottom**: AUROC). The red region of the figure is the result of standard deviation selection, and the blue region of the figure is the result of random selection. Pretraining and fine-tuning were not conducted for each test.

In this section, we present the results of the robustness of MetaSEM in different data scales. We first set the sampling points according to the standard deviation of gene expression and sample size. Then, we built sub-datasets using mHSC-L, mHSC-E, and mHSC-GM. In addition, we also created another dataset of the same points through random selection as a reference. We used the same hyperparameters and five-fold cross-validation during model training. As shown in Figure 2, the red area is larger than the blue area. The fluctuation range of the model on the three metrics was 11.5%, 15%, and 30.7%, respectively. This is because genes with a large number of 0 expression values also had a lower standard deviation. This means that removing these genes would reduce the sparsity of scRNA-seq data. By comparing the two gene selections, we found that the degree of data dispersion is an essential feature.

### 2.3. MetaSEM Reveals That GRN Specificity Is Related to Gene Expression

**Figure 3:**
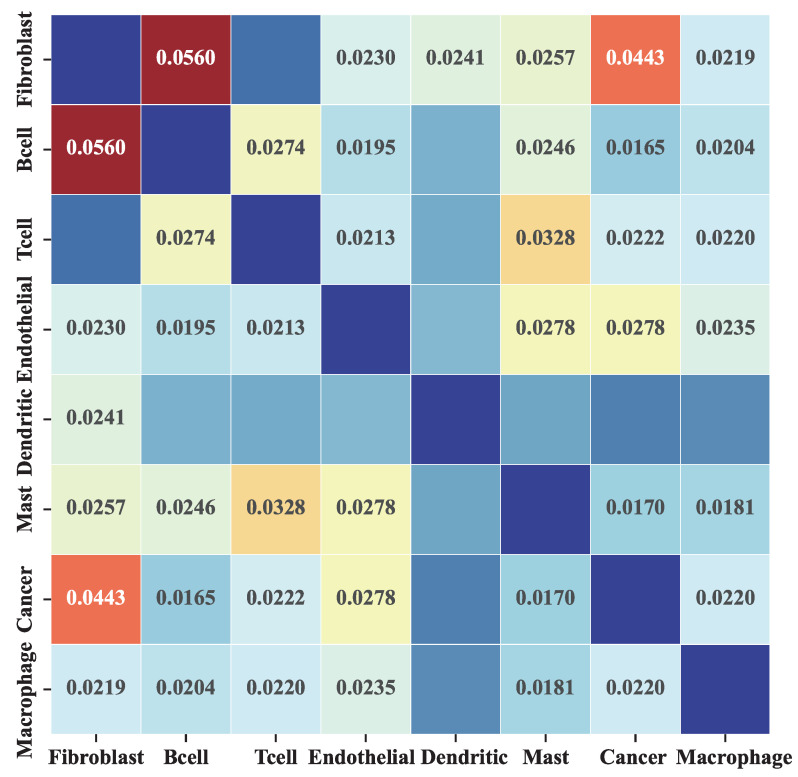
The Pearson correlation of different cell-type GRNs. Each element in the matrix represents the Pearson correlation of the GRN corresponding to two different cells. We do not show the results with a *p*-value greater than 0.05.

To verify whether MetaSEM captures specific information, we analyzed the inferred GRN and gene expression data. We generated the cell-type-specific GRN based on eight HNSCC sub-datasets. Figure 3 shows the heatmap of the Pearson correlation coefficient between the different GRNs. In this matrix, two observations are made. First, the average correlation coefficient is less than 0.1, which indicates that the inferred GRNs have a very low correlation. Second, as shown in the cancer row, the highest correlation coefficient is Fibroblast. There are two reasons for this: the cancer dataset contains some cancer-associated fibroblasts (CAFs) [24], and the correlation between Fibroblast and Cancer subsets is the highest, which corresponds to the findings of [25].

**Figure 4:**
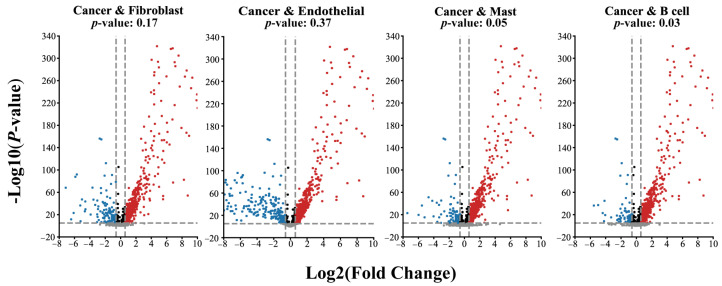
The divergence of gene expression on different cell types. The red dot represents the genes with a positive correlation, the blue dot represents the genes with a negative correlation, and the black dot represents the gene with no difference in expression level. The grey dot represents the gene below the threshold.

Given the phenomenon reflected in Figure 3, we further analyzed the differences in the gene expression in the datasets. As shown in Figure 4, the gene expression data of the Fibroblast and Endothelial subsets has little difference with the cancer subset. However, the results of the B cell and Mast subsets show significant differences. The *p*-values obtain an independent *t*-test of Fibroblast and Endothelial subsets are 0.17 and 0.37, respectively. In contrast, the Mast cell and B cell datasets are 0.05 and 0.03, respectively. These results indicate that MetaSEM can capture the specificity information, which is essential for general GRN inference tasks.

### 2.4. The Selected Regulators from the SEM Model Have a Higher Expression Level

**Figure 5:**
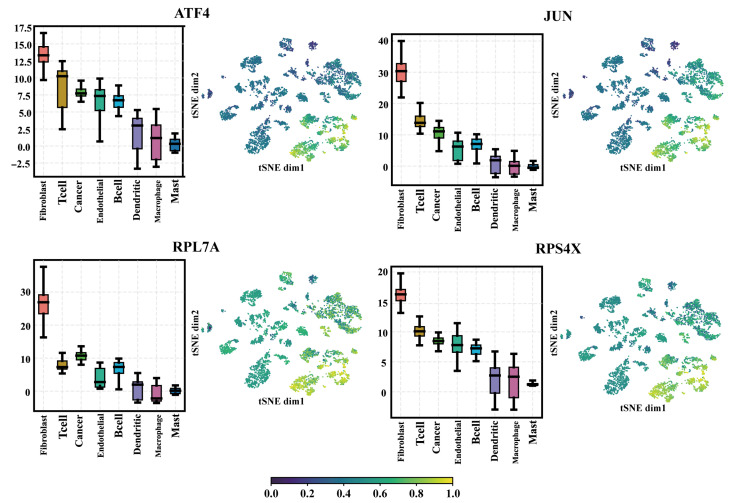
The regulatory weight of the different genes in the eight cells. Four regulators are presented: ATF4, JUN, RPL7A, and RPS4X. The boxplots show the weight distribution of the regulators on different SEM matrices. The t-SNE plots represent the weight distribution of regulators on the datasets.

Next, we analyzed how MetaSEM extracts regulators. When sorting out the output of the GRN Layer, we found that the regulatory weight of some genes was very high. Therefore, we collected the regulatory weights of these genes. The boxplots in Figure 5 show the results. These genes had higher regulatory weight in Fibroclast, T cell, Cancer, and Endothelial. As shown in the t-SNE plots of Figure 5, the selected genes had significant weight distribution on different samples. The log2 (transcripts per kilobase per million (TPM) + 1) of ATF4, JUN, RPL7A, and RPS4X on HNSCC cells were 8.1, 6.5, 6.8, and 9.8, respectively. Finally, we selected ATF4, JUN, RPL7A, RPS4X, and other genes as regulators through cross-comparison.

### 2.5. The Selected Regulators Are the Main Factors of Cell-Type Identification

**Figure 6:**
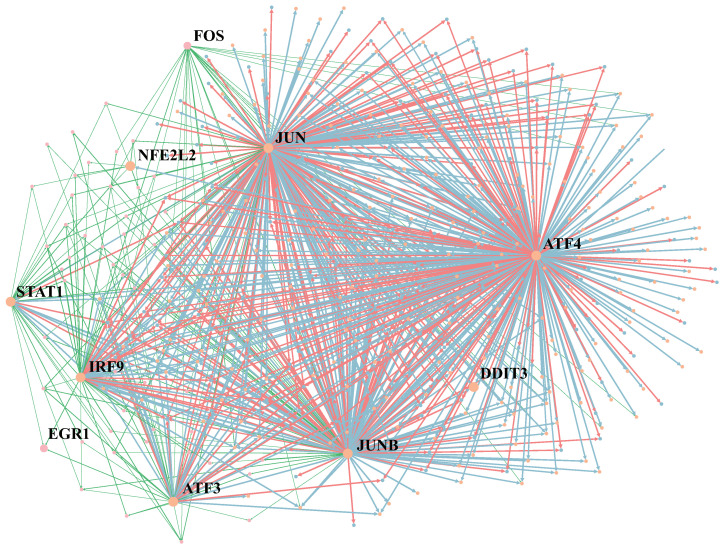
Visualization of the GRN inference by MetaSEM on Cancer and Fibroblast datasets. The size of nodes indicates the regulatory weight. The blue edges are the main part of GRN, indicating the common regulatory relationship between the two cells. Green and red regulatory relationships only exist in Cancer GRNs or Fibroblast GRNs.

In this section, we present the relationship between the selected regulators and cell-type identification. Figure 6 visualizes the GRNs of Cancer and Fibroblast datasets together. The blue edge represents the typical regulatory relationship. Green and red edges are the regulatory relationships only in Cancer GRNs or Fibroblast GRNs, respectively. We marked several regulators in GRNs, such as STAT1, JUN and JUNB. The protein encoded by STAT1 is a member of the STAT protein family. STAT1 mediates the expression of various genes, which is essential for cell viability in response to different cell stimuli and pathogens [26]. JUN and JUNB belong to the same gene family and are related to human malignancies. JUN often occurs in chromosomal regions of translocation and deletion in human malignancies [27].

**Figure 7:**
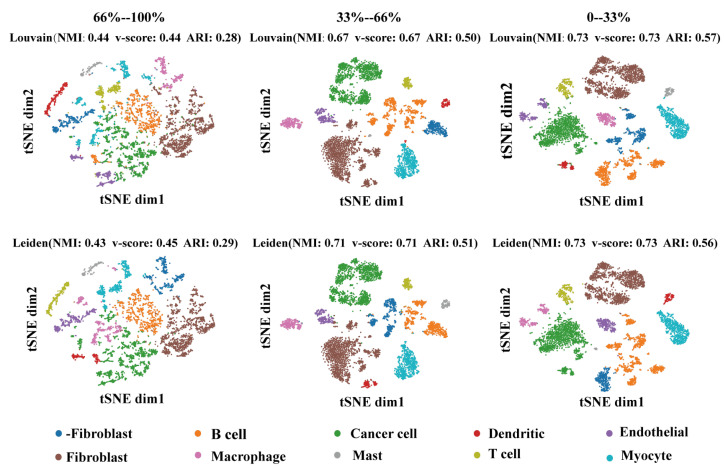
Visualization of selected regulators with different regulatory weights. Each row represents a clustering method (**top row**: Louvain, **bottom row**: Leiden). Each column represents the regulatory weight of the selected data by ascending ranking. The dimension reduction method of the graph is TSNE.

In order to verify the importance of the regulatory weight of cell-type recognition. We collected the cell types’ SEM matrix for each gene’s regulatory weight. Then, all genes were divided into three equal parts according to the regulatory weight for cell clustering. Then, we used the Louvain and Leiden methods to cluster cell types. As shown in Figure 7, the effect of clustering positively correlated with regulatory weight. The performance of the clustering methods on normalized mutual information (NMI), v-score, and adjusted rand index (ARI) proved this point.

## 3. Materials and Methods

### 3.1. Data Preparation

The BEELINE [28] dataset is used to evaluate the model’s performance. The single-cell dataset contains seven cell types, including five mouse cells and two human cells. For the BEELINE dataset, we excluded the cells annotated as low quality and genes expressed in less than 10% of cells. Then, we logarithmically normalized the remaining data. Each cell only retained the top 1000 standard deviation genes. The sub-datasets were divided according to different cell types. We further built 500 gene datasets in the same way to evaluate the model performance in insufficient label data. The ground-truth GRNs were preprocessed and normalized according to the descriptions in [28].

The head and neck squamous cell carcinoma (HNSCC) dataset [25] is used to study GRN cell specificity, a heterogeneous epithelial tumor closely related to the long-term exposure of cells to the environment of alcohol and tobacco. We divided the dataset into ten subsets based on known cell-type annotations (Fibroblast, B cell, T cell, Endothelial, Dendritic, Mast, Cancer,-Fibroblast, Myocyte, and Macrophage). We discarded the subsets without annotations, and the number of samples was less than 50. We also pitched the genes expressed in less than 30 samples. Finally, the -Fibroblast and Myocyte are removed. The genes with the top 1000 standard deviations were utilized as the training dataset. The ground-truth label corresponding to the HNSCC dataset was obtained from the TCGA database [29].

The datasets were stored in matrices with the vertical axis gene expression and the sample as the horizontal axis. One cell type corresponds to one matrix, and the values in the matrix represent the expression values of genes on the samples. Similar to DeepSEM’s training process [15], we took 64 samples of the dataset as a batch. Each batch was considered a small sample learning task, and the learning goal was unique through the loss function. There was no need to divide the test and verification sets on GRN inference.

### 3.2. Model Description

The proposed MetaSEM consisted of three parts: an encoder, a meta-decoder, and a GRN layer. (i) Encoder: This part encodes the gene expression data as the feature vector using a three-layer MLP. (ii) Meta-decoder: This part models the regulatory relationship via a two-layer MLP and uses the GRN layer to find the optimal pseudo-data labels. (iii) GRN Layer: With the SEM model, the GRN layer infers the gene regulatory relationships and transforms these relationships into pseudo-data labels.

#### 3.2.1. GRN Layer

We generalized the SEM as a GRN layer to model the conditional dependencies among random variables. We employed the meta-decoder to iterate the GRN Layer for extracting regulatory information. The final output of this module was an adjacency matrix representing GRN, and the elements within the matrix described directed edge weights. The GRN Layer iteration formula is as follows:

(1) $$A^{*}=\theta_{A}\times A+\alpha \times A$$

where *A* represents the adjacency matrix resulting from modeling based on the SEM, $\theta_{A}$ represents the model parameters of the meta-decoder, and α is utilized to control the learning rate of the matrix in the new iteration.

#### 3.2.2. Encoder

We built a decoder to capture the data feature from gene expression. The encoder reads the natural gene expression data *X* in batches. The data $X_{i}$ of batch *i* first passes through a full connection layer to learn the gene expression feature [30]. Then, the feature vector $X_{i}^{p}$ is obtained using a double-layer full connection layer.

(2) $$X_{i}^{p}=J_{F(\theta_{F})}\left(X_{i}\right)$$

where $J_{F(\theta_{F})}\left(X_{i}\right)$ represents the working process of the encoder to calculate feature vector $X_{i}^{p}$. $Y_{i}^{p}$ represents the pseudo-data label from the meta-decoder. The process obtained with the parameter of the encoder $\theta_{F}$ from each epoch can be expressed by Formula (3):

(3) $$\theta_{F}^{*}=\underset{\theta_{F}}{\arg\min}E_{v_{i}\in V^{L}}\left[L\left(J_{F(\theta_{F})}\left(X_{i}\right),Y_{i}^{p}\right)\right]$$

where *L* represents the cross-entropy loss of the network, $Y^{p}$ represents the pseudo-data label provided by the meta-decoder, and $v_{i}$ is any gene belonging to the dataset $V^{L}$.

#### 3.2.3. Meta-Decoder

We embedded the GRN Layer into a two-layer MLP to construct the meta-decoder. Unlike the attention mechanism [31,32], we employed the meta-decoder to guide the feature extraction. Specifically, the objective of the meta-decoder is to find a prediction matrix $Y_{i}^{p}$ that agrees with the label matrix *Y*. The meta-decoder learns the gene regulatory relationships from the scRNA-seq. Then, the regulatory relationships are stored in the GRN Layer. Finally, the meta-decoder outputs the pseudo-data labels to represent the potential regulatory relationships. This procedure can improve the efficiency of the encoder by pseudo-data labels.

(4) $$Y_{i}^{p}=J_{A(\theta_{A})}\left(X_{i}^{p}\right)$$

where $J_{A(\theta_{A})}\left(X_{i}^{p}\right)$ indicates the process of calculating pseudo-data labels with the meta-decoder. In each round of training, the process of calculating and updating from the feature vector set $V^{p}$ is shown in Formula (5):

(5) $$\theta_{A}^{*}=\underset{\theta_{A}}{\arg\min}E_{v_{i}\in V^{p}}\left[L\left(J_{A(\theta_{A})}\left(X_{i}^{p}\right),Y_{i}^{p}\right)\right]$$

#### 3.2.4. Hyperparameter Optimization

We employed a bi-Level optimization for hyperparameter optimization. Specifically, the bi-Level optimization calculates the model’s parameters twice and compares the gradients generated. If the gradients drop, the parameters are updated. If not, the second parameter calculation is executed again [33]. The bi-Level optimization operation can help the model approximate the optimal parameter solution, reducing the impact of the erroneous feature vector on the model. We applied a one-step gradient-descent updating strategy with a significant learning rate to obtain the approximate optimal solution. After the approximate optimal parameters are received, the model parameters need to be updated. $\mu_{\theta_{F}}$ and $\mu_{\theta_{A}}$ represent the learning rate of the encoder and meta-decoder, respectively. The parameter update operation of the encoder and meta-decoder is expressed by Formulas (6) and (7):

(6) $$\theta_{F}^{*}=\theta_{F}-\mu_{\theta_{F}}{▽}_{\theta_{F}}J_{F}\left(X_{i}\right)$$

(7) $$\theta_{A}^{*}=\theta_{A}-\mu_{\theta_{A}}{▽}_{\theta_{A}}J_{A}\left(X_{i}^{p}\right)$$

Finally, the encoder and meta-decoder outputs are extracted for model loss calculation. The overall loss calculation of the model is expressed in Equation (8):

(8) $$Loss=\underset{\theta }{\arg\min}E_{v_{i}\in V^{L}}\left[L\left(X_{i}^{p},Y_{i}^{p},:\right)+\beta \times \frac{\sum {\left(W_{1}\right)}^{2}}{2}\right]$$

where $\sum (W_{1})$ represents the weights of the first fully connected layer of the encoder. β is the weight of the encoder. Considering the feature vector and the pseudo-data labels as the main factors in the loss calculation guarantees that the contents learned by the encoder and meta-decoder remain consistent.

(9) $$X^{p*}=\theta_{F}\left(X\right)\times 0.5+X^{p}$$

Formula (9) represents the method of the encoder to the output feature vector.

### 3.3. Implementation

MetaSEM is mainly implemented based on the PyTorch framework. The determined model configurations are as follows: the encoder is built from a three-layer MLP model, and the meta-decoder is built from a two-layer MLP with the GRN Layer. Hyperparameter optimization is a one-step gradient-updating strategy based on bi-Level optimization. We employed the grid search method to determine the model’s architecture and hyperparameters. The number of hidden-layer neurons set 128, and the batch size set 64. The encoder and meta-decoder optimization employed the Adam optimizer method. The learning rate is $1\times 10^{-4}$ for the encoder, $5\times 10^{-4}$ for the meta-decoder, and $1\times 10^{-2}$ for the one-step gradient-updating strategy. In addition, we set the learning decay rate and the other parameters to default. This article presents the codes of the model and can be downloaded from GitHub.

### 3.4. Evaluation Metrics

Following the BEELINE framework [34], we applied the top *K* edges to evaluate the performance of MetaSEM on three indices: early precision ratio (EPR), Area Under the precision-recall curve (AUPR), and area under the receiver-operating characteristic curve (AUROC), where the value of *K* is equal to the number of edges of the ground-truth GRN. The above metrics were developed as indices to evaluate model performance in previous studies [10,15].

## 4. Conclusions

This paper proposed a new GRN inference algorithm based on meta-learning to analyze the importance of the selected regulators in cell-type identification. The MetaSEM learns the potential regulatory relationships from gene expression data. Moreover, meta-learning was also used to optimize the process of feature extraction. Extensive experiments in different single-cell datasets showed that MetaSEM performed better than several advanced computational methods in GRN inference tasks. Finally, by visualizing the inferred GRN, we systematically analyzed the importance of the data dispersion. We proved the importance of the selected regulator in cell-type identification. In the future, we intend to construct GRN by fusing scATAC-seq data and scRNA-seq data to explore the effect of GRNs on single cells.

## Figures and Tables

**Table 1:** Performance comparison of four competing methods on 1000 gene datasets and 500 gene datasets.

EPR										
Methods	1000 Gene Datasets							500 Gene Datasets		
	mHSC-L	mHSC-GM	mHSC-E	hESC	mESC	hHep	mDC	mHSC-L	mHSC-GM	mHSC-E
DeepSEM	1.09	1.14	1.24	1.43	1.06	1.14	2.55	1.07	1.06	1.30
DGRN	-	-	-	-	-	-	-	-	-	-
GENIE3	<1	1.03	1.01	1.00	1.06	1.12	1.01	1.00	1.06	1.03
PIDC	<1	<1	<1	<1	1.01	1.03	<1	<1	<1	<1
MetaSEM	**1.36**	**1.41**	1.24	**2.29**	**1.24**	**1.53**	**3.36**	**1.13**	**1.20**	**1.69**
**AUPR**										
Methods	1000 Gene Datasets							500 Gene Datasets		
	mHSC-L	mHSC-GM	mHSC-E	hESC	mESC	hHep	mDC	mHSC-L	mHSC-GM	mHSC-E
DeepSEM	0.63	0.56	0.42	0.30	0.38	0.48	**0.43**	0.66	0.63	0.58
DGRN	0.15	0.16	0.25	-	-	-	-	0.15	0.27	0.25
GENIE3	0.09	0.12	0.09	-	-	-	-	0.14	0.15	0.14
PIDC	0.07	0.12	0.10	-	-	-	-	0.16	0.12	0.19
MetaSEM	**0.70**	**0.73**	**0.66**	**0.48**	**0.43**	**0.70**	0.33	**0.75**	**0.77**	**0.84**
**AUROC**										
Methods	1000 Gene Datasets							500 Gene Datasets		
	mHSC-L	mHSC-GM	mHSC-E	hESC	mESC	hHep	mDC	mHSC-L	mHSC-GM	mHSC-E
DeepSEM	0.51	0.57	0.63	0.52	0.51	0.54	**0.77**	0.52	0.52	0.67
DGRN	0.63	0.67	0.75	-	-	-	-	0.63	0.71	0.77
GENIE3	0.63	0.63	0.59	-	-	-	-	0.52	0.52	0.55
PIDC	0.57	0.61	0.60	-	-	-	-	0.47	0.47	0.59
MetaSEM	**0.75**	**0.77**	**0.76**	**0.81**	**0.61**	**0.77**	0.71	**0.67**	**0.72**	**0.87**

Part of the results are from the following two papers [10,15]; “-” indicates that the experimental result is missing, and “<1” indicates that the result here is random prediction. The bold number refers to the best performer.

## Data Availability

The dataset for model training and testing is available at https://github.com/ZhangLab312/MetaSEM (accessed on 1 January 2023).
